# Antibacterial, physical and mechanical properties of bonding agent containing synthesized Zinc Dimethacrylate

**DOI:** 10.4317/jced.55636

**Published:** 2019-08-01

**Authors:** Ali Eskandarizadeh, Fahimeh Sharokhi, Faeze Hamze, Maryam Kalantari, Razieh Hoseiniffar, Mouj Khaleghi, Niloofar Shadman, Fatemeh Ramezani

**Affiliations:** 1 Kerman Social determinants on oral health research center, Kerman University of medical science, Kerman, Iran; 2 Operative department, Zahedan Dental School, Zahedan, Iran; 3 Oral and Dental disease research center, Kerman University of medical science, Kerman, Iran; 4 Chemistry Department, Faculty of Science, Shahid Bahonar University of Kerman, Kerman, Iran; 5 Operative department, Kerman Dental School, Kerman, Iran; 6 Biology Department, Faculty of Science, Shahid Bahonar University of Kerman, Kerman, Iran

## Abstract

**Background:**

The aim of this study includes synthesis of zinc dimethacrylate ionomer (ZDMA) by a new method, incorporate it into resin bonding and evaluate its antibacterial, physical and mechanical properties.

**Material and Methods:**

Resin adhesives containing 0 to 5% wt of ZDMA was produced and the following tests were accomplished: A: Antibacterial test: 1.Direct contact test. 2.Material aging; in both of them the bacterial colony counting were performed. B: Physical test: 1.Degree of conversion (D.C). 2.Evaluating the amount of released Zinc ion release in aqueous medium. C: Mechanical test: 1.Compressive strength test. 2.Shear bond test (enamel and dentine separately). The obtained results were statistically analyzed using One Way ANOVA and LSD post hoc test (α=0.05).

**Results:**

The anti-bacterial test revealed that all the ZDMA containing groups significantly reduced the amount of Streptococcus Mutans bacteria. Moreover, the D.C in all ZDMA groups was enhanced. Furthermore, ion release analysis revealed noticeable stability of Zn2+ in samples, as in the 5wt.% group it was even after nine cycle of 24h wash. On the other hand, the compressive strength was significantly reduced just in the 5% ZDMA group while the other groups were superior comparing to the control. In addition, there was no significant difference among the enamel shear bond strength of the groups. However, about the dentine shear bond strength, only the 5% ZDMA group was significantly higher than the control.

**Conclusions:**

Low percentages of ZDMA in adhesive could impart anti-bacterial efficacy without challenging its mechanical and physical properties.

** Key words:**Dental Resin Bonding, Zinc, Streptococcus mutans, Degree of conversion, Compressive strength.

## Introduction

Nowadays, tooth colored restorations are increasingly interested in dental offices according to the aesthetic demands (1,2). However, these dental resin composites have some black points that lead to shortening of their clinical service life. Secondary caries on the margins is the most important failures of dental composites, which dictate their replacement (3,4). This fact is mostly attributed to the more plaque accumulation on composite surface comparing to amalgam and glass ionomer (5), which is related to its surface roughness and free energy while they are directly affected by the resin/filler type and size (6). Actually, it has been proved that some ingredients of dental resins are metabolized by carious microorganisms (6). Therefore, establishing an anti-bacterial dental resins, which are resistant to the biofilm formation, would be most applicable for preventing these recurrent caries (7). On this subject, various approaches were accomplished to enhance the antibacterial property of resin based dental restorative materials (3,8,9). Accordingly, different antibacterial agents such as fluoride, chlorhexidine, quaternary ammonium and metal (oxide) particles/ions were physically admixed into the resinous dental materials (10-15). In addition, recently, many nano-particles were incorporated for this purpose in dental resins (2). Although nearly all of these additives impart strong anti-bacterial composites initially, their durability was reported very questionable because they would dissolve in the aqueous environment that lead to fading of anti-septic efficacy of prepared dental resin (10,16). Moreover, the time depended release of these substances from the resin matrix would results to physical voids in the composite mass that brings about lower mechanical properties of dental composites (2,10). Therefore they would adversely affect the clinical performance of tooth colored restorations in long periods especially when higher fractions of them were added (10).

Consequently, new attempts are still under investigation to achieve an efficient long lasting anti-bacterial resin for dental purpose (1). In this sense, it has been demonstrated that insoluble materials such as polyethylenimine (insoluble cross-linked quaternary ammonium) could confer durable antibacterial property to dental resin composites (9). Furthermore, employing antibacterial monomers, which are chemically bond into the resin matrix, could ensure the stability of anti-microbial activity (17,18).

As it is mentioned, metallic ions are useful anti-bacterial agents in dental materials (10). On the other hand, these ions could be chemically chelated in dental resins and encompass the aforementioned drawbacks (1). Among these metals, Zinc has an ample anti-bacterial strength and its different complexes have been frequently documented on this object in dental materials (19-23).

Zinc-methacrylate (ZM) is a functional monomer that could be copolymerized with the dental resin composite because the main backbone of most dental resins is based on methacrylate groups. Additionally, its Zinc component could impart the anti-bacterial efficacy. Hence, the ZM would be very promising for producing and identifying bactericidal tooth colored dental restorations (1).

However, very few studies were conducted on this monomer while the available data considering its cariostatic efficacy as long as its mechanical impact on dental resins are quite sparse (1). Thus, the aim of this study includes evaluating the hypothesis whether the addition of ZM into a dental resin would affect its degree of conversion and antimicrobial property. In addition the Zn 2+ ion releasing intervals were also assed.

## Material and Methods

**Figure 1:**
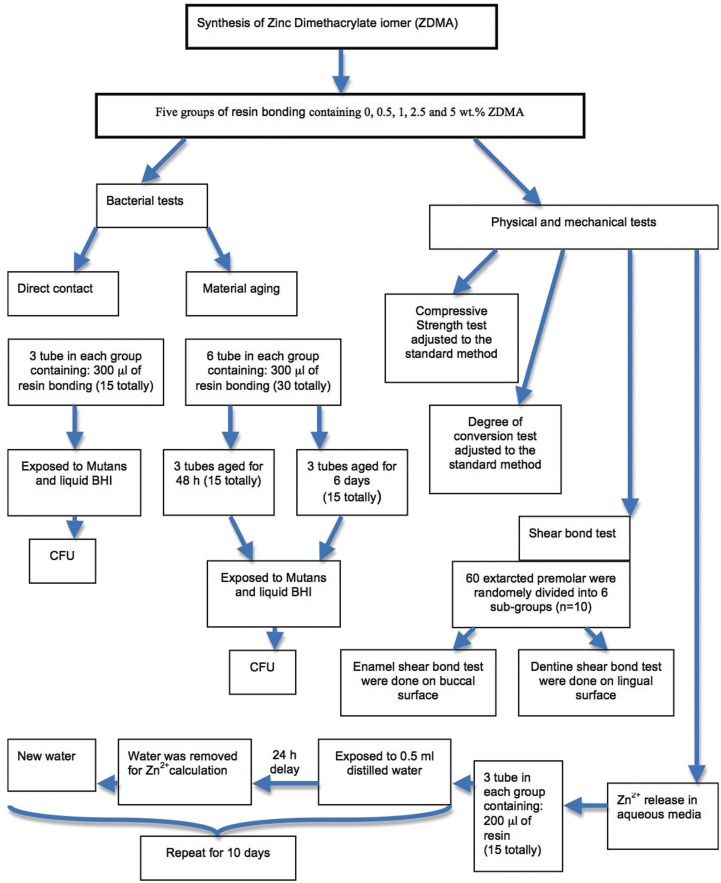
The flow chart demonstrates the summary of performed tests in the current study.

This study was approved by the local Institutional Ethics Committee (IR.KMU.REC.1395.1008). A brief presentation of the methods and materials is demonstrated in Figure 1.

-Production of Zinc Methacrylate:

100 ml of Hexan, 0.03 ml of Triton100 and 8.4 gr of ZnO were mixed with each other for 5 min at room temperature. Afterward, 17.1 gr Methacrylate acid were added to while the whole mixture were electronically shaken for 24h at 35ºC. Consequently, they were filtrated, the solvent was evaporated and the residue Zinc Dimethacrylate powder (ZDMA) were weight. Finally, the Infra Red (IR) spectroscopy was performed for confirmation of the chemical process.

-Preparation of test specimens:

In the present study, we prepared five test groups including dental resin adhesive containing ZDMA in different concentrations of 0.5, 1, 2.5 and 5 wt.% while the pure resin adhesive was considered as control group. The test groups was prepared by adding the ZDMA powder into a dental resin adhesive (Tetric N-Bond, voclar Vivodent Schaan/Liechtenstein) and homogenously mixed in a dark room for 15 min incorporating an automatic mixer . It is noteworthy that, in order to have similar conditions, although the control group was pure, the same procedure was accomplished for this group too. Finally, all the groups were stored in a completely opaque bottle prior to each test.

**Figure 2:**
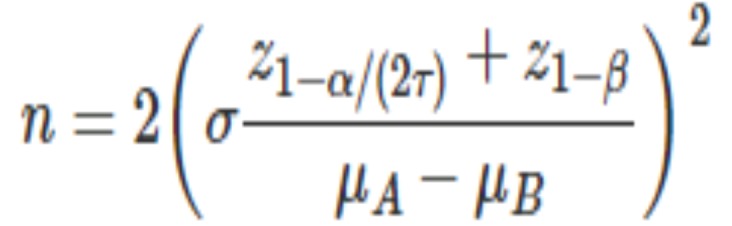
Formula.

-Sample size calculation:

For estimation of sample size, the mean and S.D value related to each performed test was obtained from the previous literature(10); while the following formula was incorporated for caculation (24): (Fig. 2).

In which the σ (S.D) and µ (mean for two groups such as group A and B) were extracted from Hojatti *et al.* study (10) in each of the performed tests including antibacterial, degree of conversion, compressive strength and bond strength tests while these data were extracted from Wassel *et al.* for ion release test (25).

Moreover, α (Type I error) was adjusted as 0.05; ß (Type II error) was consideed as 0.2 and τ (number of comparisions to be made) was 10 due to 5 sub-groups.

Beside that, the final sample size calculation was accomplished by G*Power Free Software (26,27).

-Anti-bacterial tests:

Bacterial strains and growth conditions

The standard bacteria used in this study were S. mutans PTCC 1683 (Persian Type Culture Collection, IROST, Iran). The bacteria were cultured aerobically overnight in 5 ml of brain–heart infusion (BHI) (High Media, India), at 37 ◦ C.

-Preparation of mico-titer tubes.

In order to prepare the suitable samples for anti-bacterial test, we incorporated the 500 μl, flat ended, and tightly closed mico-tubes. The tubes were vertically positioned while 300 μl of the prepared resins were purred on its flat bottom end and light cured (Demetron. Kerr, U.S.A) for 80 s by an overlapping regimen in four 20 s cycles form the top and the bottom separately. Since all the tubes had similar diameter, all the cured resin had the same surface area for future tests.

Direct Contact Test:

This test was accomplished to determine the anti-bacterial capacity of the prepared resins(10). A 10 μl of bacterial suspension (~106 bacteria) was inserted in each tube and vertically left on a bench for 1h in a sterile condition. This time interval was considered to allow for evaporation of the suspension liquid, ensuring the complete direct contact of the bacteria with the cured resin surface. Afterward, each tube was filled with 300 μl of BHI media and incubated at 37ºC. Finally, after 24 h incubation period, a uniform amount (50 μl) of this liquid mixture (bacteria + BHI broth) was cultured on blood agar plates (High Media, India and defibrinated sheep blood) for the purpose of Colony Forming Unit (CFU) Counting after 24h incubation at 37◦.

On the other hand, this test was triplicated for each group. Moreover, for controlling the whole test steps, the entire procedure was triplicatted in three empty tubes, which were served as controls.

-Material Aging:

Similar micro tubes were prepared and aged for 48h and 6 days during which the tubes were filled with 1ml of distilled water that was replaced every 48h. Ultimately, after drying of the tubes, the DCT was performed again.

Ion release test:

This test was performed in order to evaluate the longest time interval of releasing Zn2+ form the experimental resin samples in aqueous environment. For this purpose, 200 μl of each resin bonding samples containing 0, 0.05, 1, 2.5 and 5 wt.% ZDMA was inserted into the bottom end of glass tubes while they were light cured 80 sec in an overlapping regimen from the bottom of these transparent tubes. Afterward, the tubes were filled with 0.5ml of distilled water and tightly closed. In each 24h interval the distilled water was analyzed by Atomic Absorption device (SpectraAA 220, VARIAN) for measuring the Zn2+ ions and the tubes were filled with 0.5ml fresh distilled water again. This operation was repeated for ten days while we had three samples in each group (n=3).

-Degree of Conversion:

The degree of photo polymerization conversion of resin bonding specimens containing 0, 0.05, 1, 2.5 and 5 wt.% ZDMA was measured by FTIR (EQUINOX 55, Bruker, Germany) spectroscopy. The specimens were placed between two polyethylene films, pressed to form a very thin film and the absorbance peaks of the un-cured samples were obtained. The specimens were then light-cured for 40 s using the light source and the peaks were collected for the cured specimens.

**Figure 3:**
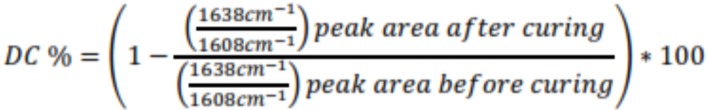
Formula.

Degree of conversion (DC%) was determined from the ratio of absorbance intensities of aliphatic C=C (peak at 1638 cm−1) against internal reference of the aromatic C•••C (peak at 1608 cm−1) before and after curing of the specimen. The degree of conversion was then calculated as follows, (Fig. 3):

For each group of resin adhesive the measurement was repeated for three times.

-Compressive Strength:

For compressive strength tests, the stainless steel cylindrical molds with diameter of 4 mm and height of 2 mm were placed on a glass slide and then over- filled with the resin bonding. After complete filling of the mold, another glass slide was pressed on the top and the whole materials cured for 40 s from the top surface. The bottom sides of the cylindrical specimens were cured for further 40 s in order to achieve higher polymerization. The compressive strength was then determined with the universal testing machine at a cross-head speed of 0.5 mm min−1. The specimens (no. = 5) were placed with their flat ends between the plates of the testing machine so that the progressively increasing compressive load was applied along the long axis of the specimens.

-Shear Bond Strength:

A total of 60 caries-free extracted human premolars were used in this in vitro study. The teeth were washed under running water immediately after extraction and stored in distilled water until the micro-shear bond strength test were performed. After perpendicular mounting, these samples were randomly divided into six groups (n=10). Consequently, prior to polishing with a 280 grit silicon carbide paper (Soft Flex, Germany), the buccal enamel surfaces were cut with a high-speed handpiece to produce a flat 2mm area perpendicular to horizontal line incorporating a surveyor. On the other hand, on the lingual surfaces the same procedure was accomplished to remove the enamel and expose at least 2mm diameter of beneath dentine. Teeth were etched with a 37% phosphoric acid solution (Total Etch 37%, Denfil, Korea) for 15 s, rinsed with water jet spray for 5 s, and air-dried. In each group, one of the five experimental resin bondings were applied on the tooth surface. Prior to light-curing, a cylinder tube (internal diameter: 1.0 mm, height: 1.0 mm) were placed on tooth surface. After 20 s irradiation using a light cure unit, each tube was carefully filled with a commercially available resin composites (Shade A1, Heliomolar, Ivoclarvivadent) and then photo polymerized for 40 s from the top of the tube. The tubes around the composite cylinders were removed by gently cutting the tube using a surgery blade, the cylindrical composite were light cured three more 40s cycles from the right, left and top sides with the purpose of being ensure about the complete polymerization. Having stored at 100% humidity at 26◦ C (room temperature), each specimen was adhered to the testing apparatus with a cyanoacrylate adhesive. A shear force was applied to the composite –tooth interface at a cross-head speed of 1 mm min−1 using the universal testing machine until failure occurred. The shear bond strength was then calculated by dividing the force at break by the composite–enamel interface area.

Data Analysis:

The obtained data were subjected to SPSS software (version 22) using One Way ANOVA and LSD post hoc tests; while the level of significance was determined as α=0.05 and the power was adjusted as 80% (ß=0.2).

## Results

**Figure 4:**
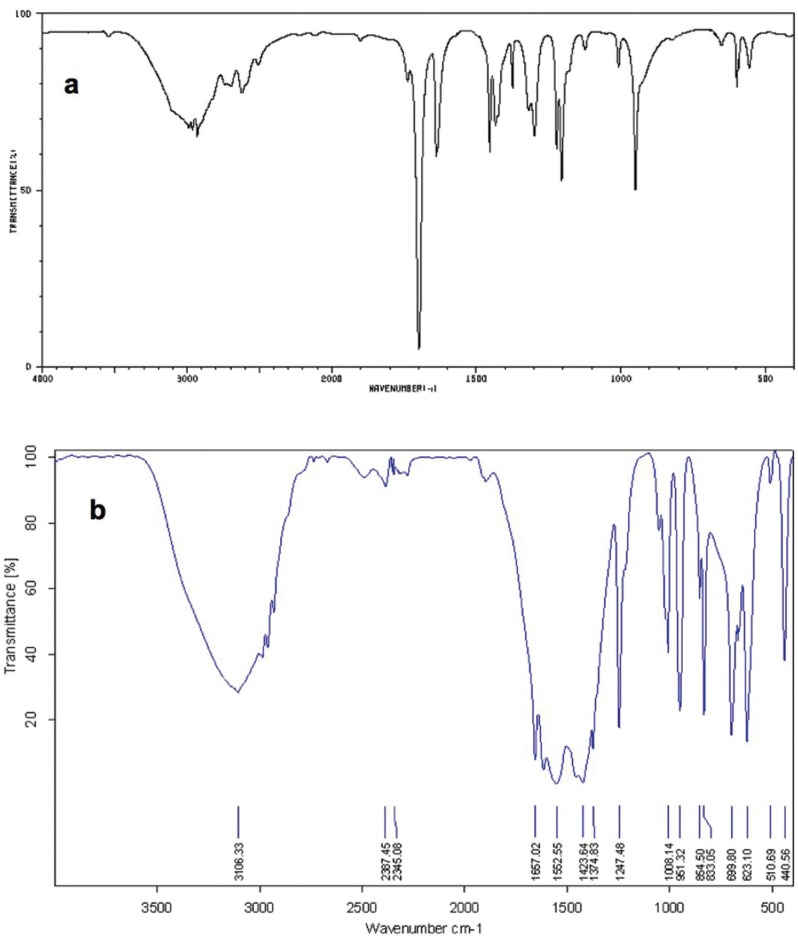
The IR diagram of the methacrylic acid (a) demonstrate the strong 1738 cm-1 band which is related to C=O resonance and the 1110 cm-1 area that is related to C-O bonds. However, after reaction (b), the C=O is shifted to 1667 cm-1 while the Zn-O absorbance band is visible around the 440 cm-1 confirming the ZDMA formation.

-Zinc Dimethacrylate production:

As it is noticeable in Fig. 4 (a & b), the C=O resonance was shifted from 1738 cm-1 to 1667 cm-1. Definitely, this shifting would confirm the formation of carboxylate anions in ZDMA. Moreover, the absorbance band related to Zn-O was observed at around 440 cm-1. Therefore, comparing the pre- and post-operation IR would confirm the production of ZDMA ionomer.

**Figure 5:**
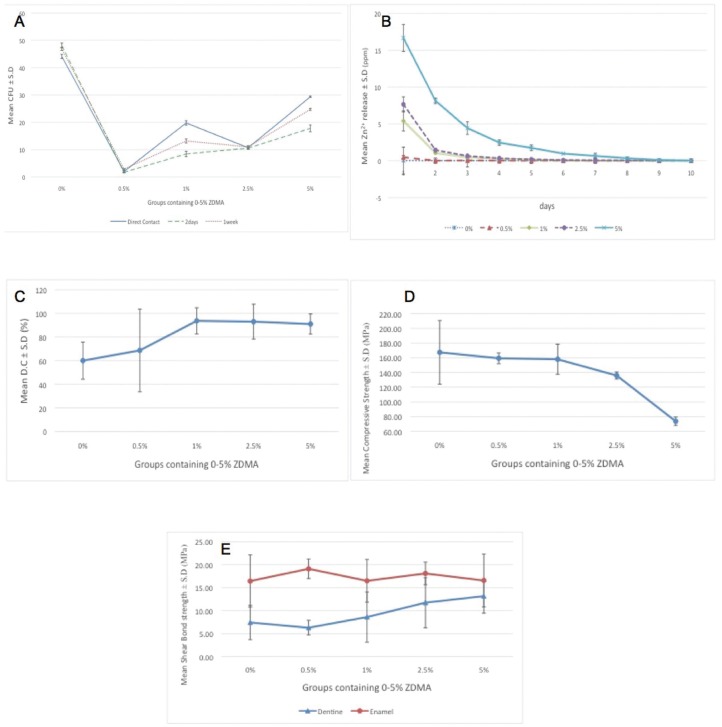
The mean ± S.D of groups (resin adhesive containing 0-5 wt.% of ZDMA) for A: Colony Forming Unit (CFU) in Direct contact and material aging tests, B: Zn2+ ion release in distilled water, C: Degree of Conversion (D.C) percentage, D: compressive strength, and E: dentine and enamel bond strengths.

-Direct contact and material aging tests:

The results of mean bacterial CFU ±SD in direct contact and material aging tests are represented in Fig. 5-A. As it can be seen, all the ZDMA containing groups suppressed the bacteria growth while the most effective group was 0.05% in all three tests intervals. One Way ANOVA revealed that the groups had statistically significant difference with each other (*P* = 0.000, 0.000 and 0.000 for direct contact, 48h and 6D in that order) and the LSD multiple comparison’s *P* values are depicted in  Table 1.

**Table 1:**
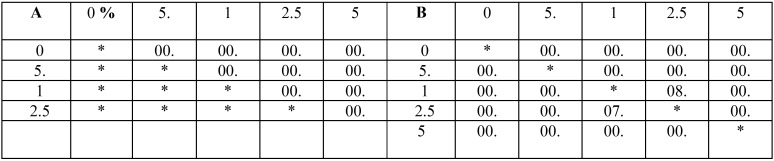
The *P* values related to LSD pair-wise comparisons of the groups in A: direct contact, and B: 2 days (above the diagonal) and 1 week (below the diagonal) bacterial test.

-Ion release:

The Fig. 5-B demonstrates the mean amount of detected Zn2+ ±SD in each group for a continuous ten days analysis. Statistical analysis revealed that in some test intervals, the five sub-groups were statistically distinguishable form each other (data related to One Way ANOVA is demonstrated in  Table 2). As can be seen, in there was no statistical significant difference among the sub-groups therefore the multiple pair wise comparisons regarding to 2nd -7th days are depicted in  Table 3. Accordingly, the stability of Zn2+ in polymer matrix was increased parallel to the amount of ZDMA content. Interestingly, these data could prove that the Zn2+ depended characteristics (such as the antibacterial property) of this experimental polymer were stable durable over several days.

**Table 2:**
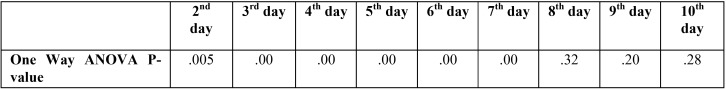
The *P* values resulted from One Way ANOVA test regarding to Zn *2*+ release in each testing interval.

**Table 3:**
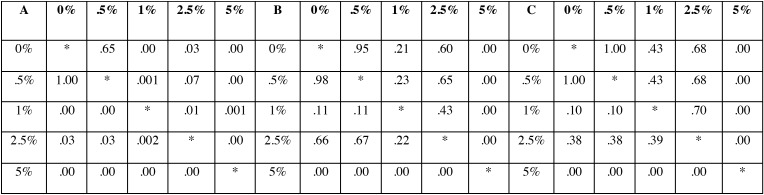
The pairwise *P* value regarding to A: 2nd day (above the diagonal) and 3rd day (below the diagonal), B: 4th day (above the diagonal) and 5th day (below the diagonal) and C: 6th day (above the diagonal) and 7th day (below the diagonal).

-Degree of Conversion:

The data that are obtained from FTIR spectroscopy are displayed in Fig. 5-C as the mean values of DC%±SD in five groups. The groups did not show any statistical significant difference with each other (*P*= 0.28). However, it is obvious, that as the ZDMA content increase in resin bonding, its DC was increased too. This is an interesting finding because it confirms the chemically bonding of synthesized ZDMA monomer into the polymeric matrix of resin bonding that would impart the superior charactristics to the polymeric backbone.

-Compressive Strength:

Fig. 5-D demonstrate the mean compressive strength ±SD of each group. There was significant difference between the groups subjecting to One Way ANOVA (*P*= 0.000) and the LSD multiple comparisions’ related *P* values are written in  Table 4. As it can be concluded from these results, the compressive strength of all ZDMA containing groups were higher than the control although only the 0.05% and 1% groups had significant difference with the unmodified samples. This finding is also quite promising because the superior mechanical behavior is always favorable in dental materials.

**Table 4:**
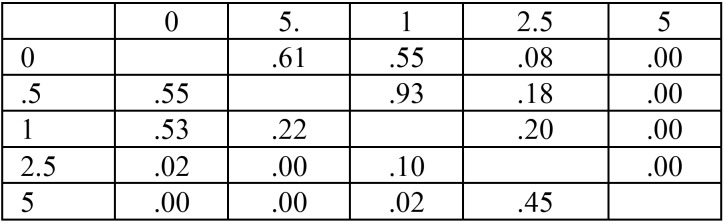
The *P* values related to LSD pair-wise comparisons of the groups in compressive strength (above the diagonal), and dentine shear bond strength (below the diagonal) tests.

-Shear Bond Strength:

The Mean±S.D value of dentine and enamel shear bond strengths are represented in Fig. 5-E.

In dentine groups, One Way ANOVA revealed significant difference among the groups (*P*=0.000), however, the LSD pair-wise P value comparisons are represented in  Table 4. Nonetheless, the mean value of dentine shear bond strength was increased as the ZDMA fraction was increase and all the ZDMA contained subgroups showed superior results comparing to the control except the 0.5% group (Fig. 5-E), but the difference was not statistically noticeable.

On the other hand, in enamel groups, there was no significant difference among neither of the subgroups (*P*=0.619). Moreover, no specific trend could be concluded in enamel mean shear bond strength by increasing the ZDMA component (Fig. 4-E).

## Discussion

The results of the current study revealed strong anti-bacterial effect of ZDMA containing resin bonding against S.mutans (the main etiology for dental caries).

This finding is in accordance with Henn S *et al.* (1) who admixed the Zinc Methacrylate (ZM) with their experimental resin bonding. However, they reported the anti-bacterial effect in 10, 20 and 30% of ZM while in lower concentrations of ZM they did not observe any suppressive effect against S.mutans. This difference could be attributed to the micobiological methodology since we used the DCT whilst they incorporated agar diffusion method and they measured the inhibition zone hallow around their sample (1). Although the agar diffusion test is the most famous method for evaluation of the antimicrobial capacity of various materials (9,28), its mechanism is based on the water solubility of the substance . Therefore, for solid mass especially the resinous components, which are not leachable in the aqueous media, the agar test would have a bias on the microbial results and DCT would be preferable because via this process the bacteria would be exposed directly to the material and their feasibility of proliferation would be assessed (10,29-31). It is noteworthy that after DCT in BHI media, we incorporate the CFU to obtain the number of viable bacteria while in some other previous researches the turbidity of the liquid culture were evaluated by means of spectrophotometry (32). We selected the CFU counting because in this method the live and the dead bacteria would be distinguished from each other (32,33). In contrast, in the spectrophotometry all the bacteria regardless of their viability would be considered (32,33). Obviously, the active and vital S.mutans have the main responsibility for dental caries and they should be recognized from the dead ones to show the practical anti-bacterial efficacy of a dental material (32,33).

On the other hand, in order to estimate the sustainability of Zn2+ in the polymerized adhesive, the aging (anti-bacterial test after one week) as well as the ion release tests were performed. Both of the mentioned tests confirmed the durability of Zn2+ within the adhesive. Actually, the anti-bacterial activity of our samples was continued after three washing cycles (six days time period). In the previous studies on ZM containing adhesive, the stability of the antimicrobial effect was not reported (1). However, in literatures, which physically admixed the Zinc Oxide nano-particles with resin, the anti-bacterial capacity was not persistent after one week (three washing cycles) (10). Interestingly, our atomic spectroscopy revealed that even after nine washing sequence, the Zn2+ would be released from the cured 5 wt.% content ZDMA adhesive media, so our persistent anti-bacterial effect was confirmed. Accordingly, it could be concluded that in oral environment the anti-bacterial capacity of ZDMA containing adhesive would be considered more beneficial clinically comparing to the physically admixed Zn components.

The promising role of ZDMA in the adhesive resin was also confirmed by our DC% test in which the DC of the ZDMA containing samples was higher than the unmodified resin. Since the higher DC% would endow superior mechanical behavior of the polymers (34), this phenomenon could reflect the significant advantage of ZDMA addition into dental adhesive. Moreover, our DC% outcome is completely similar to Henn S *et al.* who reported the increased DC% in ZM groups (1). However, in their study there was no difference among groups containing 1 to 30% ZM that is completely parallel to our results. Nevertheless, our mean DC% value is below the previous researches on Tetric N-Bond adhesive. The laboratory process of mixing the commercially available adhesive with the ZDMA could explain this strange finding. Although all the protective strategies such as working in a dark environment and cooling of the dishes was considered to decrease the chance of solvent evaporation and environmental lighting, maybe some carbon double bonds were converted to single bonds prior to FTIR testing. Thus the whole percent of carbon double bond conversion was diminished in the resin matrix.

Our results revealed that the ZDMA had favorable effect on dentine shear bond strength that could be related to the chemical interaction between the Zn2+ and the collagen. This finding is in accordance with Toledano et al. who investigated the dentine bond strength of some dental adhesives containing ZnO and they reported superior results after three months comparing to the control while after 24h they did not report any significant difference among the groups (35). However, they admixed the ZnO physically by the resin adhesive and they argued that in the three months perid the Zn was inhibit the collagen degradation comparing to the conrol. However, our Zn containg particle was different from their study , So we observed superior results even after 24h. Whilst, the addition of ZDMA did not have any significant effect on enamel bonding that was a predictable result since the ZDMA does not have any interaction with enamel structure (which is mostly consists of hydroxyapatite crystals). This fing was parallel to the Hojati *et al.* who physically admixed the ZnO nano-particles with dental resin adhesive (10).

Overwhelmingly, our investigation revealed the beneficial effect of ZDMA in dental resin bondings; however more studies are strongly suggested.

## Conclusions

Incorporation 0.5 to 2.5 wt.% of ZDMA into dental resin bonding would enhance its anti-bacterial effect without compromising the physical and mechanical charactristics. Moreover, interestingly, addition of up to 5wt. % of the mentioned ionomer into dentine bonding would enhance its shear bond strength to dentine.
